# Can Foot Orthoses Benefit Symptomatic Runners? Mechanistic and Clinical Insights Through a Scoping Review

**DOI:** 10.1186/s40798-024-00774-w

**Published:** 2024-10-04

**Authors:** Francis Del Duchetto, Cloé Dussault-Picard, Martine Gagnon, Philippe Dixon, Yosra Cherni

**Affiliations:** 1 École de Kinésiologie et des Sciences de L’activité Physique, Université de Montréal, Montréal, QC Canada; 2 Laboratoire de Neurobiomécanique & Neuroréadaptation de la Locomotion (NNL), Centre de Recherche Azrieli du CHU Ste Justine, Montréal, QC Canada; 3 Département de Kinésiologie, Faculté de Médecine, Université Laval, Québec, QC Canada; 4 Department of Kinesiology and Physical Education, McGill University, Montréal, QC Canada; 5 Centre Interdisciplinaire de Recherche sur le Cerveau et L’apprentissage (CIRCA), Montréal, QC Canada; 6 Institut de Génie Biomédical, Université de Montréal, Montréal, QC Canada

**Keywords:** Foot sole, Running, Overuse injury, Pain, Biomechanics, Insoles

## Abstract

**Background:**

Running is a widely practiced sport worldwide associated with a host of benefits on cardiovascular, metabolic, musculoskeletal, and mental health, but often leads to musculoskeletal overuse injuries. The prescription of a foot orthosis (FO) is common to manage musculoskeletal impairments during physical activity or functional tasks. Although FOs are frequently prescribed by clinicians for symptomatic populations of runners, the existing literature supporting the prescription of FOs in runners has predominantly focused on either uninjured individuals or a mix of uninjured and symptomatic populations. Thus, the effects of FOs on the treatment and/or prevention of overuse running injuries need to be investigated to guide future research and assist clinicians in their decision-making process.

**Main body:**

This scoping review aimed to evaluate the immediate and long-term effects of FOs on lower limb biomechanics, neuromuscular parameters, and pain and disability in symptomatic runners, and to identify factors that may influence the effects of FOs. Five databases (CINAHL, SPORTDiscus, MEDLINE, Embase, and Web of Science) were searched, resulting in 2536 studies. A total of 30 studies, published between 1992 and 2023 (730 symptomatic runners), were included following the removal of duplicates and the screening process. Wearing FOs while running is related to an immediate and a long-term decrease in pain and symptoms of overuse running injuries. Also, wearing FOs while running decreases eversion at the foot/ankle complex, leads to a more lateral plantar pressure at the heel and forefoot, and may change running motor control strategies. Finally, the effectiveness of FOs is influenced by its added features.

**Conclusions:**

This study provides recommendations for future research such as the need for standardized methods in describing FOs, considering participant characteristics such as foot morphology, and comparing different types of FOs. Also, this scoping review provides valuable insights for guiding the prescription and design of FOs, and suggests that integrating FOs into a comprehensive treatment plan may yield better results than standalone first-line treatments. Nonetheless, this scoping review highlights the need for future research to explore the optimal integration of FOs into injury-specific treatment plans.

**Supplementary Information:**

The online version contains supplementary material available at 10.1186/s40798-024-00774-w.

## Key Points

- Foot orthoses may redistribute loads onto non-injured structures, thereby resulting in an immediate decrease in pain and potentially contributing to the treatment of injuries.
- A rearfoot medial wedge decreases rearfoot and/or calcaneal eversion motion and moments in injured runners.
- A foot orthosis integrated into a comprehensive treatment plan may yield better results compared to standalone first-line treatments.
- The use of sham/flat insoles as a control/blinding condition is crucial in evaluating the effect of a foot orthosis.

**Supplementary Information:**

The online version contains supplementary material available at 10.1186/s40798-024-00774-w.

## Background

Running is a widely favoured sport worldwide with an ever-increasing rate of participation [1, 2]. It is associated with a range of benefits such as better cardiovascular [3], metabolic [4], skeletal [5], and mental health [6], as well as a decrease in all-cause mortality risk [3]; however, running often leads to musculoskeletal overuse injuries [7], especially in novice and recreational runners. These populations experience up to 33 running injuries per 1000 h of running with medial tibial stress syndrome (MTSS), Achilles tendinopathy (AT), plantar fasciitis (PF), patellofemoral pain syndrome (PFPS) or anterior knee pain (AKP), iliotibial band syndrome (ITS), and ankle sprains (AS) being most prevalent [7–9]. In addition to pain and disability [10], injuries often result in adverse effects including negative psychosocial impacts [11], a decreased participation in physical activity [12], and a loss of productivity in daily-living tasks [13].

To treat and manage lower extremity musculoskeletal pathologies or injuries, clinicians frequently prescribe foot orthoses (FOs) [14, 15]. In general terms, a FO is a device inserted between the plantar aspect of the foot and the shoe, intended to treat or manage injuries or pathologies of the lower limb [16–18]. The different types of FOs can be classified according to their materials, hardness, rigidity, purpose, and manufacturing methods [19–21]. However, due to variations in fabrication and prescription methods among different countries and professionals [20, 22, 23], there is no universal classification for FOs. Experts often divide FOs into three categories: (1) simple foot orthosis (SFO), which consist of a flat cushioning insole that can be customized with added features such as a valgus (lateral) or varus (medial) wedge, an arch support, and a metatarsal dome; (2) prefabricated foot orthosis (PFO), which is an insole designed based on generic foot morphology, with arch contouring, and can be customized with the same features as SFOs and/or through heat mouldings; and (3) customized foot orthosis (CFO), which is manufactured from a 3D impression or a computerized image of the patient’s feet [20, 21, 24, 25].

FOs are employed to manage musculoskeletal impairments during physical activity [26] and functional tasks [27], yielding varying effects [28]. Although their effects are not fully understood, their therapeutic properties primarily stem from direct mechanical effects [26, 29, 30], neuromuscular modulation [31, 32], and somatosensory changes [33–35]. In runners, FOs are mainly used to treat and/or prevent overuse injuries and increase running performance [36]. While two systematic reviews have reported low [37] to moderate [38] quality evidence supporting the use of FOs in preventing overuse running injuries, no review has been published regarding the use of FOs in the treatment of such injuries. These findings collectively contribute to a lack of understanding regarding the effects of FOs on the treatment and/or prevention of overuse running injuries.

To address this gap, some reviews have examined the effect of wearing FOs on running biomechanics [30–32, 39–41]. Although FOs are frequently prescribed by clinicians for symptomatic populations of runners [42], existing reviews have predominantly focused on either uninjured individuals [31, 41] or a mix of uninjured and symptomatic populations [32, 39, 40]. Additionally, no review has examined the long-term effects of wearing FOs, overlooking the chronicity and repetitive nature of overuse injuries in runners. Therefore, further investigation into both the immediate (while running, without a period of adaptations) and long-term (while running, following a period of adaptations) effects of wearing FOs is necessary to better understand their utility in the clinical management of symptomatic runners. Finally, no review has addressed the effect of the type of FOs on reported biomechanical outcomes, representing a fundamental gap in choosing FO prescription based on literature knowledge.

Thus, this scoping review aimed to (1) evaluate the immediate and long-term effects of FOs on lower limb biomechanics (*i.e.*, kinematics, kinetics, plantar pressure and force), neuromuscular parameters (*i.e.*, muscle activity), and pain and disability in symptomatic runners; and (2) identify factors that may influence the effects of FOs (*i.e.*, types of FO, injury location, intervention duration) to guide future research and assist clinicians in their decision-making process. The PICO question and details are presented in Supplementary material 1.

## Methods

### Protocol and Search Strategy

This scoping review was conducted following the criteria of the Preferred Reporting Items for Systematic reviews and Meta-Analyses extension for Scoping Reviews (PRISMA-ScR) checklist [43], and in accordance with the methodological steps established by Arksey and O’Malley [44]. The search protocol was elaborated with the initial help of a health science librarian from Université Laval (Québec, Canada). The literature search was conducted on 5 databases: CINAHL (EBSCO), SPORTDiscus (EBSCO), MEDLINE (OVID), Embase (ELSEVIER), and Web of Science (CLARIVATE). The search strategy was based on two main concepts: FO and running. The keywords and scripts stemming from these concepts were adapted to each database. The protocol was registered on the OSF registry platform (DOI: https://doi.org/10.17605/OSF.IO/7TXK2).

### Study Selection

A study was included if the following criteria were met: (1) original cross-sectional or longitudinal intervention study; (2) study targeting symptomatic and/or injured adult human runners; (3) study reporting at least one immediate or long-term effect of wearing FO on running (*e.g*., kinematics, kinetics, electromyography (EMG), plantar pressure and force, pain, comfort, injury symptoms); and (4) study with full text available in English or French. A study was excluded if (1) only simultaneous interventions were conducted (*e.g.*, FOs and physiotherapy), and/or a mixed population (*e.g.*, injured and non-injured) was included, leading to the inability to isolate the effects of FO during running, (2) if the FO were worn in footwear other than running shoes (*e.g*., military boots, soccer shoes), (3) if kinematics data were not acquired using a camera-and-marker based motion analysis system, and (4) if the running task was exclusively sprinting and/or the participants were exclusively sprinters.

### Study Screening

First, all articles identified by the database search were transferred to Covidence (https://www.covidence.org/), and duplicates were removed. Second, titles and abstracts were screened independently by two authors (FDD and YC), based on the inclusion criteria. Third, the selected articles were full text reviewed by the same authors (FDD and YC). In cases of unresolvable disagreement related to the selection or elimination of a study, a third author (CDP) established a consensus.

### Data Charting Process and Analysis

Data were extracted and organized into tables and charts based on (1) general information: title, year of publication, author names, study design, (2) methodological information: population characteristics of the intervention and control groups (*i.e.*, the number of participants, age, biological sex, disability and symptoms, foot type, and running volume), intervention details (*i.e*., type of FO, FO materials and customization method, protocol and data collection details, and outcomes assessed), and (3) study results concerning significant effect (*i.e*., immediate or long-term) of FO compared to not wearing FO or to placebo version of a FO such as a sham/flam insole in the symptomatic population. Immediate effects were considered if the participant did not receive any adaptation time to the FO, or if the adaptation period was too short based on intervention practices, *i.e*., less than 2 weeks [45]. Long-term effects were considered for studies that reported differences occurring after 3 weeks of wearing the FO. Data extraction was conducted by one author (FDD) and validated by two other authors (YC & CDP).

The results were summarized through the application of both descriptive and numerical analyses. Effect sizes (ES) of each statistically significant effect (*p* < 0.05) of wearing FO during running (with FO vs. without FO) were reported. If the original study did not provide the ES, it was calculated from the mean and standard deviation data. The authors were contacted if the mean and standard deviation were not available. For the studies that used parametric tests, a normal distribution of the data was assumed, and a Cohen’s d ES (*d*) was calculated [46]. Otherwise, a Glass’s delta ES (△) was calculated [47]. ES below 0.2 were considered very small, 0.2–0.5 as small, 0.5–0.8 as medium, 0.8–1.0 as large, and those above 1.0 as very large effects [48].

### Methodological Quality and Risk of Bias

The quality of the included studies was assessed using the modified version of the Downs and Black checklist [49], which has been developed to assess the methodological quality of randomized and non-randomized studies of health care interventions. Considering the broad range of study and protocol design, all items were deemed relevant by the authors. However, the items pertaining to the follow-up of participants were not scored for studies with only one data acquisition session (items 9, 17, 26) and certain items concerning the selection bias were not scored for studies with only one group of participants (items 21, 22, 23, 24). The studies were assigned a quality score of “high” (≥ 75%), “moderate” (60–74%), 22 “low” (≤ 60%) [29]. The study quality of three randomized articles was first assessed by two authors (FDD and CDP) to ensure standardization of the evaluation method. Then, all articles were assessed by two authors (FDD and CDP). A third author (YC) resolved any disagreement.

## Results

### Search Results

**Fig. 1:**
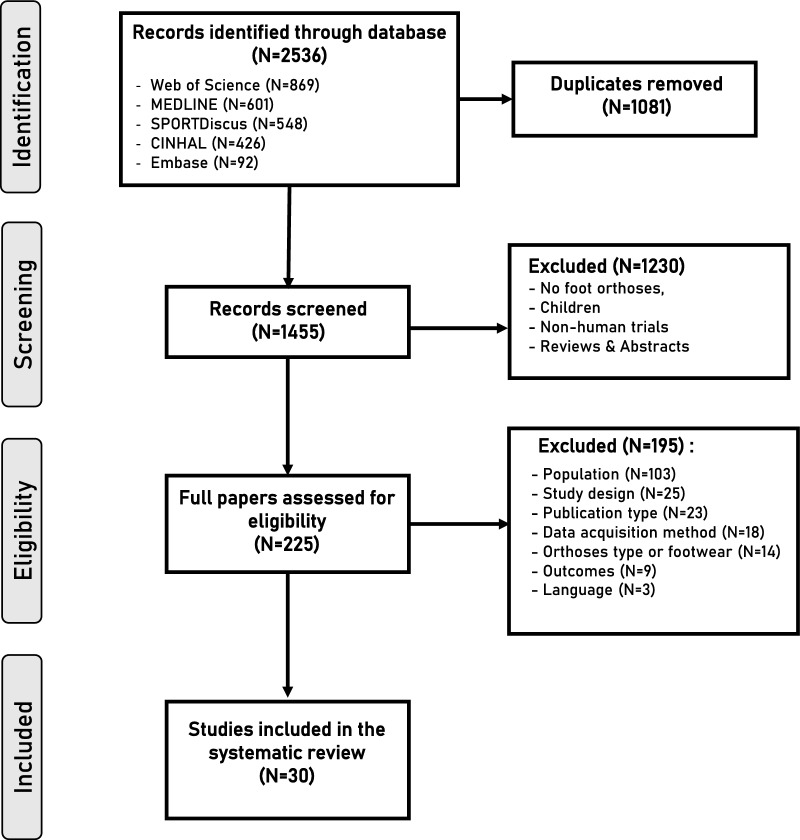
PRISMA flowchart of the study selection process

The initial search resulted in 2536 studies. After removing duplicates (n = 1081), 1455 studies were screened which led to the inclusion of 30 studies. The flowchart of the selection process is shown in Fig. 1.

### Risk of Bias

Based on the modified Downs & Black checklist, the overall median score of the included studies was 67% (ranged from 33 to 100%), indicating a moderate quality (see Table 1). From these, 10 studies were assessed as high quality [50–59], 12 were of moderate quality [60–71], and 8 were of low quality [72–79]. The omission of reporting adverse effects (item 8) and the absence of blinding of both participants and researchers were the principal limitations (items 14 and 15). Only 4 of 30 (13%) studies took measures to blind the participants, either with sham/flat insoles [57, 65, 68] or by not informing them about the variation of different FOs [54], whereas 4 studies (13%) made an effort to blind the researchers [50–52, 57]. Additionally, only 7 studies (23%) sought to report adverse events linked to the wearing of FOs, such as blisters, new injuries, and other major discomforts [51–54, 57, 58, 66]. Of note, the study by Naderi et al. [57] was scored 100%, which was verified by a third author (YC).

**Table 1:** Methodological quality assessment scores of included studies using the modified version of Downs and Black checklist

Items		Studies																													
		49	50	59	51	71	60	61	72	52	53	62	54	73	63	64	55	56	65	74	75	66	67	68	69	76	77	78	57	58	70
Reporting	1	1	1	1	1	1	1	1	1	1	1	1	1	1	1	1	1	1	1	1	1	1	1	1	1	1	1	1	1	1	1
	2	1	1	1	1	1	1	1	1	1	1	0	1	1	1	1	1	1	1	1	1	1	1	1	1	1	1	1	1	1	1
	3	1	1	1	1	0	0	1	1	1	1	1	1	0	1	1	1	1	0	0	0	1	1	1	1	0	1	0	1	1	1
	4	0	1	1	0	1	0	0	0	1	1	1	1	1	0	0	0	1	1	1	1	1	1	1	1	0	0	1	0	1	1
	5	2	2	2	2	0	2	2	1	2	2	2	2	0	1	1	2	2	2	1	0	2	2	2	2	1	1	2	2	2	2
	6	1	1	1	1	1	1	1	1	1	1	1	1	1	1	1	1	1	1	1	1	1	1	1	1	1	1	1	1	1	1
	7	1	0	0	0	0	1	0	0	0	1	0	1	0	1	0	1	1	0	0	0	0	0	1	0	0	0	0	1	1	1
	8	0	1	0	1	0	0	0	0	1	1	0	0	0	0	0	0	1	1	0	0	0	0	0	0	0	0	0	1	0	0
	9	1	1	▲	1	▲	▲	▲	▲	1	1	1	▲	▲	1	▲	▲	1	▲	▲	▲	▲	1	1	▲	▲	▲	▲	▲	▲	▲
	10	1	1	1	1	0	1	1	1	0	1	1	1	1	0	1	1	1	0	1	1	1	1	0	0	0	1	1	1	1	0
External validity	11	1	1	1	1	*	1	1	1	1	1	1	1	1	1	1	1	1	1	1	1	1	*	*	1	1	*	*	1	1	1
	12	1	1	1	1	*	1	1	1	1	1	1	1	1	1	1	1	1	1	1	1	1	*	*	1	1	*	*	1	1	1
	13	1	1	1	1	1	1	1	1	0	1	1	1	1	1	1	1	1	1	1	*	1	*	1	1	1	1	1	1	1	1
Internal validity—bias	14	0	0	0	0	0	0	0	0	0	1	0	0	0	0	1	0	1	0	0	0	0	1	0	0	0	0	0	0	0	0
	15	1	1	0	1	0	0	0	0	0	0	0	0	0	0	0	0	1	0	0	0	0	0	0	0	0	0	0	0	0	0
	16	1	1	0	1	0	1	1	1	1	1	0	1	1	1	1	1	1	1	1	1	0	1	1	1	1	1	1	1	1	1
	17	1	1	▲	1	▲	▲	▲	▲	1	1	1	▲	▲	1	▲	▲	1	▲	▲	▲	▲	1	1	▲	▲	▲	▲	▲	▲	▲
	18	1	0	0	0	0	1	0	0	0	1	0	1	0	1	0	1	1	0	0	0	0	0	1	0	0	0	0	1	1	1
	19	1	1	1	0	1	1	0	1	1	1	0	1	1	0	1	1	1	1	1	1	1	0	0	1	1	1	1	1	1	1
	20	1	1	1	1	1	1	1	1	1	1	1	1	1	1	1	1	1	1	1	1	1	1	1	1	1	1	1	1	1	1
Internal validity—confounding	21	1	1	*	1	▲	0	▲	0	1	1	▲	▲	*	1	1	1	1	1	▲	▲	1	*	▲	▲	1	▲	▲	1	▲	▲
	22	1	1	*	1	▲	1	▲	1	1	*	▲	▲	1	1	1	1	1	1	▲	▲	1	*	▲	▲	1	▲	▲	1	▲	▲
	23	1	1	0	1	▲	0	▲	0	1	1	▲	▲	0	1	0	0	1	0	▲	▲	0	1	▲	▲	0	▲	▲	0	▲	▲
	24	1	1	0	1	▲	0	▲	0	1	1	▲	▲	0	1	0	0	1	0	▲	▲	0	1	▲	▲	0	▲	▲	0	▲	▲
	25	1	1	1	1	0	0	1	0	0	1	1	0	0	0	0	1	1	0	0	0	1	1	1	1	1	0	0	1	0	0
	26	1	1	▲	1	▲	▲	▲	▲	1	1	1	▲	▲	1	▲	▲	1	▲	▲	▲	▲	1	1	▲	▲	▲	▲	▲	▲	▲
Power	27	1	1	1	0	0	0	1	1	1	1	1	1	1	0	0	1	1	0	0	0	0	0	0	0	0	1	0	0	0	0
Score (%)	89	89	60	79	33	60	67	56	75	93	67	81	52	68	60	76	100	60	57	48	64	61	67	67	52	52	52	76	76	71	
Quality	H	H	M	H	L	M	M	L	H	H	M	H	L	M	M	H	H	M	L	L	M	M	M	M	L	L	L	H	H	M	

Study number is based on the reference number within the text. The score for each item (except for item 5) is 1 = Yes, 0 = No, * = Unable to determine, ▲ = not applicable. The score for item 5 is: 2 = Yes, 1 = partially, and 0 = No. The global quality is noted by H = High, M = Moderate, and L = Low

### Study Characteristics

**Fig. 2:**
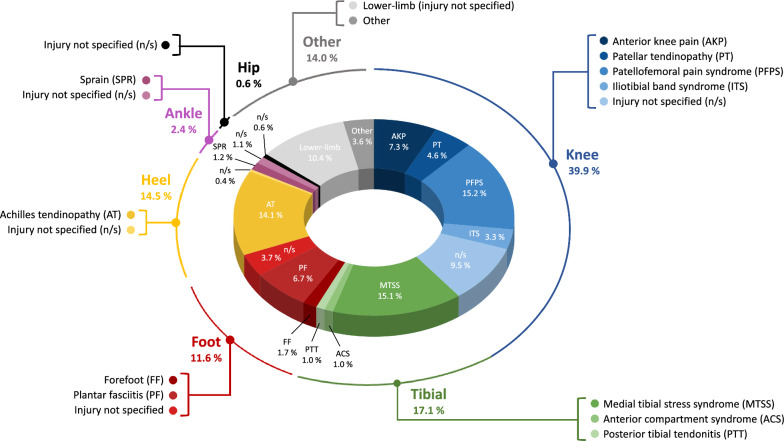
Distribution of injuries or pain among symptomatic populations in the included studies

The studies included were published between 1992 and 2023. A total of 730 symptomatic runners (58.6% women) were included. Table 2 presents the population characteristics of the included studies. The sample size ranged from 7 to 81 (mean ± SD of 24.3 ± 19.1) with a group mean age of 31.4 ± 6.3 years old, if reported. Individuals ran more than 12.9 km/week in the 17 studies (57% of the included studies) that reported this information. For 110 participants from 7 different studies, the injury or the sport-related symptoms were not specified. For the other participants, the knee was the most common pain-related location (n = 314, 17 studies), followed by the tibial (n = 135, 7 studies), heel (n = 114, 7 studies), foot (n = 91, 8 studies), ankle (n = 19, 3 studies), and hip (n = 5, 1 study) (see Fig. 2).

**Table 2:** Summary of the general and population information of the included studies

Study	Population					
	n	Age years old (SD)	Sex	Disability and symptom Injury or pain location	Foot morphology characteristic (tool or criteria to assess)	Running volume during the study duration km/week (SD)
Andreasen et al. [50]	SG1: 14 SG2: 20	SG1: 43.0 (2.8) SG1: 41.0 (3.8)	SG1: 2 M, 12F SG2: 1 M, 19F	Foot = SG1: 5, SG2: 5 AT = SG1: 2, SG2: 2 FF = SG1: 9, SG2: 4 Ankle = SG1: 4, SG2: 3 Heel = SG1: 2, SG2: 1 PTT = SG1: 2, SG2: 4 PF = SG1: 6, SG2: 8 MTSS = SG1: 3, SG2: 2	SG1 and SG2: Excessive pronation (standing calcaneal valgus angle > 6°)	n/m
Baur et al. [51]	SG1: 39 SG2: 42	SG1: 37.1 (8.3) SG2: 37.3 (8.2)	50 M, 49F	AT = SG1: 14, SG2: 12 PT = SG1: 8, SG2: 10 PFPS = SG1: 7, SG2: 7 ITS = SG1: 7, SG2: 6 PF = SG1: 3, SG2: 4 MTSS = SG1: 3, SG2: 4 Other = SG1: 6, SG2: 8	n/m	SG1: 43.7 (21.3) SG2: 44.1 (23.4)
Boldt et al. [60]	SG: 20	SG: 21.3 (2.6)	SG: 20F	PFPS = SG: 20	SG: excessive pronation n = 7/20 (standing calcaneal posture)	SG: 15.6 (8.1)
Bonacci et al. [52]	SG: 7	SG: 34.0 (9.5)	SG: 4 M, 4F	PFPS = SG: 7	n/m	SG: 15.6 (6.6)
Dixon and McNally [72]	SG: 22	n/m	n/m	AT = SG: n/m AKP = SG: n/m PF = SG: n/m LBP = SG: n/m MTSS = SG: n/m	n/m	SG: > 32.2
Donoghue et al. [61]	SG: 12	SG: 37.8 (8.1)	SG: 11 M, 1F	AT = SG: 12	SG: Excessive pronation (podiatrist judgment: qualitative analysis of barefoot running)	n/m
Donoghue et al. [62]	SG: 12	SG: 38.7 (8.1)	SG: 11 M, 1F	AT = SG: 12	SG: Excessive pronation (podiatrist judgment: qualitative analysis of barefoot running)	n/m
Ferber et al. [73]	SG: 11	SG: 29.9 (12.2)	SG: 5 M, 6F	PF = SG: 4 PFPS = SG: 2 PTT = SG: 1 ACS = SG: 4	Excessive pronation (physical therapist: static visual assessment)	n/m
Hirschmüller et al. [53]	SG1: 39 SG2: 42	SG1: 37.1 (8.3) SG2: 37.3 (8.2)	50 M, 49F	AT = SG1: 14, SG2: 12 PT = SG1: 8, SG2: 10 PFPS = SG1: 7, SG2: 7 ITS = SG1: 7, SG2: 6 PF = SG1: 3, SG2: 4 MTSS = SG1: 3, SG2: 4 Other = SG1: 6, SG2: 8	n/m	SG1: 43.7 (21.3) SG2: 44.1 (23.4)
Lewinson et al. [54]	SG1: 13 SG2: 14	SG1: 28.6 (8.7) SG2: 33.6 (9.9)	SG1: 6 M, 7F SG2: 5 M, 9F	PFPS = SG1: 13, SG2: 14	n/m	SG1: 15.1 (7.5) SG2: 21.3 (9.9)
MacLean et al. [63]	SG: 12	SG: 18.0—35.0	SG: 12F	Knee = SG: 12	n/m	SG: 15.0—40.0
MacLean et al. [55]	SG: 12	SG: 19.0—35.0	SG: 12F	Knee = SG: 12	n/m	SG: 15.0—40.0
MacLean et al. [74]	SG: 9	n/m	SG: 9F	Knee = SG: 9	n/m	n/m
Mayer et al. [64]	SG1: 8 SG2: 9	SG1: 38.0 (4.9) SG2: 35.0 (6.7)	SG1: 8 M SG2: 9 M	AT = SG1: 8, SG2: 9	n/m	SG1: 53.1 (10.6) SG2: 50.0 (13.5)
Mills et al. [65]	SG1: 27 SG2: 13	SG1: 28.7 (6.1) SG2: 31.2 (4.4)	SG1: 19F, 8 M SG2: 10F, 3 M	AKP = SG1: 27, SG2: 13	SG1: Mobile foot SG2: Less mobile foot (change in midfoot width between weight and non-weight bearing)	n/m
Naderi et al. [56]	SG: 50	SG: 21.9 (2.4)	SG: 50 M	MTSS = SG: 50	SG: supinated n = 2/50, normal n = 12/50, pronated n = 34/50 (FPI)	SG: 15.7 (2.7)
Naderi et al. [57]	SG1: 25 SG2: 25	SG1: 25.5 (5.5) SG2: 27.1 (6.2)	SG1: 25F SG2: 25F	MTSS = SG1: 25, SG2: 25	SG1 and SG2: Low arch (Dynamic arch index ≥ 26%)	SG1: 13.6 (3.5) SG2: 14.3 (3.2)
Nawoczenski et al. [66]	SG: 20	SG: 30.2 (9.2)	SG: 11 M, 9F	Hip = SG: 5 Knee = SG: 6 Leg = SG: 3 Ankle = SG: 2 Foot = SG: 12	SG: Pes planus or low arch n = 10, Pes cavus or high arch n = 10 (radiography)	n/m
Nawoczenski and Ludewig [75]	SG: 12	SG: 27.2 (9.9)	SG: 6 M, 6F	Hip, knee, leg, ankle, or foot = SG: 12	SG: At least one of the criteria: Tibial varum of > 5°, non-weight-bearing RF varus deformity of > 5°, non-weight-bearing FF varus deformity of > 5°, first ray excessive mobility, and at least 2 of the criteria: Lateral calcaneal inclination angle ≤ 20°, lateral talometatarsal angle ≤ -4°, anterior–posterior talometatarsal angle ≤ -2°. (radiography)	n/m
Orteza et al. [76]	SG: 10	SG: 17.0 (3.1)	SG: 7 M, 3F	Acute inversion ankle sprain (≤ 6 weeks) = SG: 10	n/m	n/m
Rodrigues et al. [67]	SG: 33	SG: 31.9 (9.2)	SG: 11 M, 22F	AKP = SG: 17	n/m	SG: > 12.9
Shih et al. [68]	SG1: 12 SG2: 12	SG1: 34.4 (9.8) SG2: 31.3 (8.3)	18 M, 6F	Knee = SG1 and SG2: 21 Foot = SG1: 2, SG2: 1	SG1 and SG2: Excessive pronation (△NH > 10 mm, non-weight-bearing RF varus > 5°, weight-bearing calcaneal valgus > 5°)	SG1: 19.1 (10.8) SG2: 25.0 (17.5)
Sinclair et al. [69]	SG: 17	SG: 34.1 (10.4)	SG: 10 M, 7F	PFPS = SG: 17	SG: Neutral (n/m)	SG: 17.3 (8.4)
Sinclair and Butters [70]	SG: 17	SG: 33.1 (8.4)	SG: 17 M	PFPS = SG: 17	n/m	SG: ≥ 35.0
Stell and Buckley [77]	SG: 30	SG: 28.3 (9.0)	SG: 14 M, 16F	n/m	Excessive pronation (biomechanical examination)	n/m
Van Lunen et al. [78]	SG: 17	SG: 36.2 (16.2)	5 M, 12F	PF = SG: 17	n/m	n/m
Williams III et al. [79]	SG: 11	SG: 30.6 (11.4)	SG: 5 M, 6F	PTT = SG: 1 PF = SG: 4 ACS = SG: 4 PFPS = SG: 2	n/m	n/m
Wyndow et al. [58]	SG: 15	SG: 42.0 (7.0)	SG: 15 M	AT = SG: 15	SG: Neutral (FPI score: 2.0 ± 3.0)	SG: ≥ 20.0
Zhang et al. [59]	SG: 15	SG: 25.0 (5.0)	SG: 8 M, 7F	Knee = SG: 15	Excessive pronation (FPI score: 7.8 ± 1.3)	SG: 20.3 (8.3)
Zhang and Vanwanseele [71]	SG: 12	SG: 25.8 (5.5)	SG: 7 M, 5F	Lower-leg = SG: 15	Excessive pronation (FPI score: 7.9 ± 1.4)	SG: 19.9 (7.9)

Abbreviations: ACS, anterior compartment syndrome; AKP, anterior knee pain; AT, Achilles tendinopathy; F, female; FF, forefoot; FPI, foot posture index; ITS, iliotibial band syndrome; LBP, lower-back pain; M, male; MTSS, medial tibial stress syndrome; NH, navicular height; n/m, not mentioned; PF, plantar fasciitis; PFPS, patellofemoral pain syndrome; PT, patellar tendinopathy; PTT, posterior tibial tendonitis; SD, standard deviation; SG, symptomatic group; RF, rearfoot

Table 3 presents the methodological characteristics of the 30 included studies. Among them, 13 studies (43%) assessed immediate effects of FOs [56, 59, 60, 65, 67, 68, 70–72, 76–79], 14 studies (47%) focused on long-term effects (*i.e.*, after wearing FOs more than 3 weeks) of FOs [50, 52, 53, 55, 57, 58, 61, 62, 64, 66, 69, 73–75] and 3 studies (10%) assessed both immediate and long-term effects of FOs [51, 54, 63]. Studies that reported immediate effects were conducted immediately after the participant received the FOs (*i.e*., the same day), except for two studies that completed the data collection after 2 weeks of use [68, 77]. Long-term effects were assessed after 10.6 ± 12.3 weeks (range (min to max): 3 to < 52 weeks). One study did not specify how long the participants wore their FOs [62], but mentioned that FOs were prescribed for low-grade AT, and all participants were asymptomatic at the time of testing. The effects of FOs were assessed by comparing running with and without FO for the same symptomatic group in 16 studies (53%) [52, 55, 56, 58, 60–63, 66, 67, 69, 70, 72, 74, 75, 78], a symptomatic group that ran in various FO conditions in 6 studies (20%) (*i.e*., variation of one type of FO [59, 71, 73, 79], different types of FOs such as CFO versus SFO [77], and/or flat control FO [76]), two symptomatic groups (one group with FOs, one group without FOs) in 4 studies (13%) [50, 51, 53, 64], or two symptomatic groups that ran in various FO conditions in 4 studies (13%) (*i.e*., variation of one type of FO [54, 65] and/or flat control FO [57, 65, 68]). Overall, 15 studies (50%) assessed the effects of CFOs [50, 51, 53, 55, 61–64, 66, 72–76, 79], 10 studies (33%) assessed the effects of PFOs [52, 56–59, 65, 69–71, 78], 4 studies (13%) assessed the effects of SFOs [54, 60, 67, 68], and only one study (3%) compared two different types of FOs (CFO versus SFO) (see Table 3, Supplementary materials 2 and 3) [77]. Furthermore, 4 studies (13%) used a flat sham as controls [57, 65, 68, 76].

**Table 3:** Summary of the methodological information of the included studies

Study	Methodology			
	Assessed conditions FO type worn, added features & specifications	Protocol Number of data collection sessions (week)	Data collection Condition, running speed, duration, or number of trials/conditions	Outcomes assessed Variables reported (tool)
Andreasen et al. [50]	SG1: Personal footwear SG2: CFO, n/a	3 sessions (0, 16, and 52 weeks)	n/a	Pain & symptom: Overall pain (VAS)
Baur et al. [51]	SG1: Neutral footwear SG2: CFO, 3-mm FF lateral wedge and 25-mm arch support	2 sessions (0 and 8 weeks)	Treadmill, imposed speed (3.3 m/s), 20 gait cycles	Muscle activity: Peroneus longus time activation and amplitude (Ambu surface electrodes)
Boldt et al. [60]	SG: 1- Study footwear 2- SFO, 6° full-length medial wedge	1 session (0 week)	20-m overground, S-SS (3.5–3.9 m/s), 5 trials	Kinematic: Hip and knee internal rotation and adduction (8-Eagle digital cameras) Kinetic: Hip and knee abudction moment (8-Eagle digital cameras, Bertec force plate)
Bonacci et al. [52]	SG: 1- Personal footwear 2- PFO, 6° medial wedge	2 sessions (0 and 12 weeks)	n/a	Pain & symptom: Overall pain (VAS), anterior knee pain (numeric scale), global improvement (numeric scale)
Dixon and McNally [72]	SG: 1- Study footwear 2- CFO, n/a	1 session (0 week)	8-m overground, imposed speed (3.8 ± 5% m/s), 10 trials	Kinematic: RF eversion, ankle dorsiflexion, tibial internal rotation, knee flexion (VICON cameras) Plantar pressure and force: Peak lateral and medial heel and foot balance occurrence times (RSscan pressure plate)
Donoghue et al. [61]	SG: 1- Personal footwear 2- CFO, medial wedge < 10°	1 session (< 52 weeks)	Treadmill, S-SS (2.8 ± 0.3 m/s), 1 min	Kinematic: Ankle dorsiflexion, knee flexion, RF eversion, calcaneal angle, leg abduction (8-Qualisys ProReflex cameras)
Donoghue et al. [62]	SG: 1- Personal footwear 2- CFO, medial wedge < 10°	1 session (n/m)	Treadmill, S-SS (2.8 ± 0.3 m/s), 1 min	Kinematic: Ankle dorsiflexion, knee flexion, RF eversion, calcaneal angle, leg abduction (8-Qualisys ProReflex cameras)
Ferber et al. [73]	SG: 1- Study footwear 2- CFO, 4° RF intrinsic medial wedge 3- CFO, inverted	1 session (16 weeks)	25-m overground, self-selected (3.7 ± 5% m/s), 8 trials	Kinematic: Joint coupling angle and variability of RF and tibial rotation (6-VICON cameras)
Hirschmüller et al. [53]	SG1: Neutral footwear SG2: CFO, FF lateral wedge	2 sessions (0 and 8 weeks)	n/a	Pain & symptom: Overall pain (PES)
Lewinson et al. [54]	SG1: SFO, 6-mm full-length medial wedge SG2: SFO, 3-mm full-length lateral wedge (Study footwear)	2 sessions (0 and 6 weeks)	30-m overground, S-SS (4.0 ± 0.2 m/s), 5 trials	Pain & symptom: Patellofemoral pain (VAS) Kinetic: KAAI (8-Motion Analysis Corp. cameras, Kistler AG force plate)
MacLean et al. [63]	SG: 1- Study footwear 2- CFO, 5° RF intrinsic medial wedge	2 sessions (0 and 6 weeks)	20-m overground, S-SS (4.0 ± 5% m/s), 5 trials	Pain & symptom: Knee symptoms (numeric scale), knee pain (numeric scale) Kinematic: RF and calcaneal eversion, tibial adduction, ankle eversion, knee rotation, tibial rotation, femoral rotation, knee adduction, knee flexion (8-Qualisys ProReflex cameras) Kinetic: Ankle inversion moment and impulse, knee external rotation, abduction, and extension moment and impulse, impact, loading rate (8-Qualisys ProReflex cameras, AMTI force plate)
MacLean et al. [55]	SG: With or without: CFO, 5° RF intrinsic medial wedge 1- Soft shoe midsole 2- Medium shoe midsole 3- Hard shoe midsole	1 session (6 weeks)	~ 6-m overground, S-SS (4.0 ± 5% m/s), 5 trials	Kinematic: RF and calcaneal eversion, tibial and femoral internal rotation (8-Qualisys cameras) Kinetic: Ankle inversion and knee external rotation moment and impulse, vertical impact, loading rate (8-Qualisys cameras, AMTI force plate)
MacLean et al. [74]	SG: 1- Study footwear 2- CFO, 5° RF intrinsic medial wedge	1 session (6 weeks)	Treadmill, S-SS (3.0 ± 5% m/s), 30 min	Kinematic: Intralimb coupling for tibia rotation and calcaneus eversion, knee flexion and RF eversion, knee abduction and RF eversion, knee rotation and RF eversion (8-Qualisys cameras)
Mayer et al. [64]	SG1: Personal footwear SG2: CFO, FF lateral wedge, arch support	2 sessions (0 and 4 weeks)	Treadmill, imposed speed (80% anaerobic threshold), 20 min	Pain & symptom: Pain during activity (PES)
Mills et al. [65]	SG1 and SG2: 1- PFO, n/m 2- PFO, n/m 3- PFO, n/m 4- sham/flat insole (Personal footwear)	1 session (0 week)	Treadmill, S-SS (2.7 ± 1.8 m/s), 3 min	Kinematic: Pelvis, hip, and knee flexion–extension, internal–external rotation, and adduction-abduction, ankle plantar-dorsiflexion and foot eversion-inversion (14-VICON cameras) Muscle activity: Peak amplitude and temporal derivatives (onset, offset and time to peak) of TA, SOL, MG, RFE, VL, VM, BF, GM (Viasys NeuroCare surface electrodes)
Naderi et al. [56]	SG: 1- Personal footwear 2- PFO, 25-mm peak-height arch support	1 session (0 week)	12-m overground, S-SS (3.3 ± 5% m/s), n/m	Plantar pressure and force: Total contact time, first metatarsal contact time, FF flat and heel off time, and relative first contact time and end of contact, peak pressure, and absolute impulse of 10 anatomical areas (RSscan pressure plate)
Naderi et al. [57]	SG1: sham/flat insole SG2: PFO, 25-mm peak-height arch support (Personal footwear)	4 sessions (0, 6, 12, and 18 weeks)	n/a	Pain & symptom: MTSS symptoms (MTSS scale)
Nawoczenski et al. [66]	SG: 1- Personal footwear 2- CFO, n/a	1 session (3–4 weeks)	Treadmill, S-SS, 2 min	Kinematic: Tibial internal–external rotation, calcaneal abduction–adduction (3-Panasonic 450AG cameras)
Nawoczenski and Ludewig [75]	SG: 1- Study footwear 2- CFO, n/a	1 session (3–4 weeks)	Treadmill, S-SS, 2 min	Muscle activity: Mean RMS amplitude of TA, MG, VL, VM, BF during the loading phase (silver-silver chloride electrodes)
Orteza et al. [76]	SG: 1- Personal footwear 2- CFO, n/a 3- sham/flat insole	1 session (0 week)	18-m overground, S-SS, n/m	Pain & symptom: Perceived pain (numeric scale)
Rodrigues et al. [67]	SG: 1- Study footwear 2- SFO, 4° RF and FF medial wedges	1 session (0 week)	Treadmill, imposed speed (2.9 m/s), 30 s	Kinematic: RF eversion angle and velocity, RF abduction–adduction, peak tibial internal rotation, knee internal rotation (8-Qualisys cameras)
Shih et al. [68]	SG1: sham/flat insole SG2: SFO, 5° RF medial wedge (Personal footwear)	3 sessions (0, 1, and 3 weeks)	Treadmill, S-SS, 60 min	Pain & symptom: Overall pain (VAS), pain onset (time)
Sinclair et al. [69]	SG: 1- Personal footwear 2- PFO, n/a	2 sessions (0 and 4 weeks)	Overground, S-SS (4.0 ± 5% m/s), n/m	Pain & symptom: Knee pain (KOOS-Patellofemoral scale) Kinematic: Knee and ankle flexion–extension, abduction–adduction, inter-external rotation, and tibial internal–external rotation (8-Qualisys cameras) Kinetic: Patellofemoral force, knee adduction moment (Kistler force plate)
Sinclair and Butters [70]	SG: 1- Personal footwear 2- PFO n/a	1 session (0 week)	20-m overground, S-SS (4.0 ± 5% m/s), 5 trials	Kinematic: Knee and ankle flexion–extension, abduction–adduction, inter-external rotation, and tibial internal–external rotation (8-Qualisys cameras) Kinetic: Patellofemoral force, knee adduction moment (Kistler force plate)
Stell and Buckley [77]	SG: 1- Personal footwear 2- CFO, n/a 3- SFO, 5° RF wedge and arch support	1 session (2 weeks)	Treadmill, S-SS (3.3–4.0 m/s), ~ 20 steps	Kinematic: RF pronation angle and velocity, calcaneal eversion and velocity, calcaneal angle at heel strike
Van Lunen et al. [78]	SG: 1- Personal footwear 2- PFO, 6° RF medial wedge	1 session (0 week)	Overground, S-SS (2.2–3.1 m/s), 1.5 min	Pain & symptom: Heel pain (VAS scale) Plantar pressure and force: Lateral and medial RF and FF plantar pressure (Pedar in-shoe pressure system)
Williams III et al. [79]	SG: 1- Study footwear 2- CFO, 4° RF medial wedge 3- CFO, inverted	1 session (n/m)	25-m overground, S-SS (3.4 ± 10% m/s), 5 trials	Kinematic: RF eversion, knee adduction, and tibial and knee internal rotation (6-VICON cameras) Kinetic: RF inversion moment, power, and negative work, and knee abduction moment (BERTEC force plate)
Wyndow et al. [58]	SG: 1- Study footwear 2- PFO, n/a	1 session (6 weeks)	25-m overground, S-SS (4.0 ± 10% m/s), 6 trials	Muscle activity: Onset and offset timing of SOL, MG, LG (Meditrace 100 surface electrodes)
Zhang et al. [59]	SG: 1- Study footwear 2- PFO, 4-mm medial FF wedge, 20-mm arch support 3- PFO, 2-mm medial FF wedge, 20-mm arch support 4- PFO, 20-mm arch support 5- PFO, 2-mm lateral FF wedge, 20-mm arch support 6- PFO, 4-mm lateral FF wedge, 20-mm arch support 7- PFO, 4-mm medial FF wedge, 24-mm arch support 8- PFO, 2-mm medial FF wedge, 24-mm arch support 9- PFO, 24-mm arch support 10- PFO, 2-mm lateral FF wedge, 24-mm arch support 11- PFO, 4-mm lateral FF wedge, 24-mm arch support	1 session (0 week)	20-m overground, S-SS, 8 steps	Plantar pressure and force: Contact time, COP trajectories, and force time integral under hallux, 2nd metatarsal, 3rd metatarsal, 5th metatarsal, and medial heel (Rscan pressure plate)
Zhang and Vanwanseele [71]	SG: 1- Study footwear 2- PFO, 4-mm medial FF wedge, 20-mm arch support 3- PFO, 2-mm medial FF wedge, 20-mm arch support 4- PFO, 20-mm arch support 5- PFO, 2-mm lateral FF wedge, 20-mm arch support 6- PFO, 4-mm lateral FF wedge, 20-mm arch support 7- PFO, 4-mm medial FF wedge, 24-mm arch support 8- PFO, 2-mm medial FF wedge, 24-mm arch support 9- PFO, 24-mm arch support 10- PFO, 2-mm lateral FF wedge, 24-mm arch support 11- PFO, 4-mm lateral FF wedge, 24-mm arch support	1 session (0 week)	Treadmill, S-SS (2.2 ± 0.2 m/s), 2 min	Kinematic: FF dorsiflexion, abduction, and eversion, and RF dorsiflexion, external rotation, and eversion (13-VICON cameras)

Abbreviations: BF, biceps femoris; CFO, customized foot orthosis; COP, center of pressure; EVA, ethylene vinyl acetate; FF, forefoot; FO, foot orthosis; GM, gluteus maximus; KAAI, knee abduction angular impulse; KOOS, knee injury and osteoarthritis outcome score; LG, lateral gastrocnemius; MG, medial gastrocnemius; MTSS, medial tibial stress syndrome; n/a, not applicable; n/m, not mentioned; PDI, Pain disability index; PES, pain experience scale; PFO, prefabricated foot orthosis; RF, rearfoot; RFE, rectus femoris; SG, symptomatic group; SFO, simple foot orthosis; SF-36, short-form health survey; SOL, soleus; S-SS, self-selected speed; TA, tibialis anterior; VAS, visual analog pain score; VL, vastus lateralis; VM, vastus medialis

A total of 16 studies (53%) focussed on running kinematics [55, 60–63, 65–67, 69–74, 77, 79], 7 studies (23%) on running kinetics [54, 55, 60, 63, 69, 70, 79], 4 studies (13%) on plantar pressure and force [56, 59, 72, 78], 4 studies (13%) on muscle activity [51, 58, 65, 75], and 13 studies (43%) reported the effects of FOs on pain and symptoms [50, 52–54, 57, 63–65, 68–70, 76, 78]. Among the 26 studies (87%) that assessed the effects of FOs during running in a laboratory setting [51, 54–56, 58–79] 12 studies (46%) were conducted on a treadmill [51, 61, 62, 64–68, 71, 74, 75, 77] and 14 studies (54%) overground [54–56, 58–60, 63, 69, 70, 72, 73, 76, 78, 79]. A total of 22/26 studies (85%) were conducted with a self-selected speed (mean speed range: 2.2–4.0 m/s for the 18/22 studies that reported the self-selected average speed) [54–56, 58–63, 65, 66, 68–71, 73–79], whereas 4/26 studies (15%) imposed the running speed (mean speed range: 2.9–3.7 m/s for the 3/4 studies that mentioned the imposed speed) [51, 64, 67, 72].

### The Effect of FOs on Running Kinematics

**Fig. 3:**
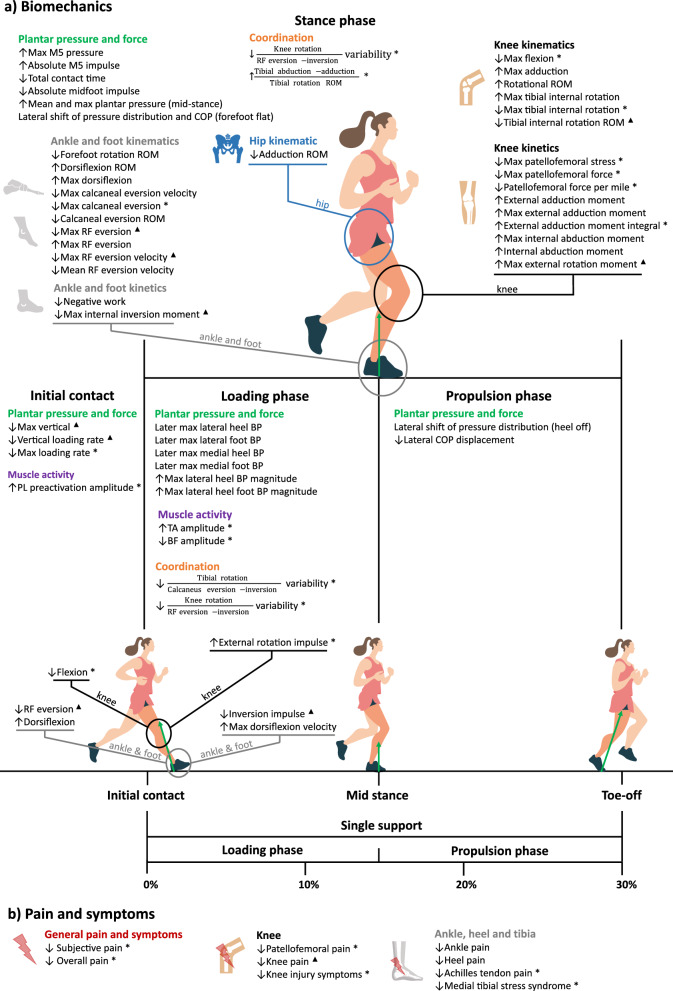
Effects of wearing a foot orthosis compared to not doing so on running. **a** biomechanics during the stance phase, and **b** pain and symptoms. All significant results reported in Tables 4 and 5 are presented. If available, the effects of a foot orthosis are presented for the subphase-specific occurrence across stance phase (initial contact, loading phase, or propulsion phase). For each effect, an asterisk (*) denotes a long-term effect, a black triangle (**▲**) indicates a long-term and immediate effect, and no symbol represents an immediate effect. ABD, abduction; ADD, adduction; BP, balance pressure; COP, center of pressure; M5, fifth metatarsal; PL, peroneus longus; QoL, quality of life; RF, rearfoot; ROM, range of motion; TA, tibial anterior; ↑, increased; ↓, decreased

*Hip.* Only one study reported an immediate effect of wearing FOs while running on hip kinematics [60], namely a reduction in adduction range of motion (ES = 0.218–small effect) (see Table 4, Fig. 3a).

**Table 4:** Immediate and long-term effects of wearing a foot orthosis compared to not doing so on running kinematics and kinetics

Kinematics								
Hip kinematics			Knee and tibial kinematics			Ankle and foot kinematics		
**FO effect (sub-phase)**	**S**	**ES (I/L)**	**FO effect (sub-phase)**	**S**	**ES (I/L)**	**FO effect (sub-phase)**	**S**	**ES (I/L)**
↓ADD ROM	59	0.218 ^I^	↑ Rotational ROM ↑Max ADD ↓Flexion (IC) ↓Max flexion ↓Tibial internal rotation ROM ↑Max tibial internal rotation ↓Max tibial internal rotation	66 78 68 68 69, 65 78 54	0.150 ^I^ 0.390 ^I^ 0.510* ^L^ 0.280* ^L^ 0.191 ^I^, 0.414 ^L^ 0.400 ^I^ 0.480 ^L^	↓FF rotation ROM ↑Dorsiflexion (IC) ↑Max dorsiflexion ↑Dorsiflexion ROM ↑Max dorsiflexion velocity (LP) ↓Calcaneal eversion ROM ↓Max calcaneal eversion ↓Max calcaneal eversion velocity ↓RF eversion (IC) ↓RF eversion ROM ↓Max RF eversion ↑Max RF eversion ↓Mean RF eversion velocity ↓Max RF eversion velocity	70 71 71 70 69 76 54 76 71, 66, 61 76, 66 62, 66, 71, 62 60, 61 71 71, 62, 66, 76, 54, 62	• ^I^ 0.277 ^I^ 0.187 ^I^ • ^I^ 0.267 ^I^ 1.213 ^I^ 0.590 ^L^ 1.638 ^I^ 0.352 ^I^, 0.650 ^I^, 0.315* ^L^ 1.334 ^I^, 0.360 ^I^ • ^I^, 0.810 ^I^, 0.500^I^, •^L^ 1.010^I^, 0.642*^I^ 0.387 ^I^ 0.276 ^I^, • ^I^, 0.710 ^I^, 2.247 ^I^, 0.740 ^L^, • ^L^
**Coordination**								
**FO effect (sub-phase)**	**S**	**ES (I/L)**						
↓ $\frac{\text{Tibial rotation}}{\text{Calcaneus eversion}-\text{inversion }}\text{variability }(\text{LP})$ ↓ $\frac{\text{Knee rotation}}{\text{RF eversion}-\text{inversion }}\text{variability }(\text{LP})$ **↓** $\frac{\text{Knee rotation}}{\text{RF eversion}-\text{inversion }}\text{variability}$ ↑$\frac{\text{Tibial abduction}-\text{adduction}}{\text{Tibial rotation}}$	73 73 73 65	0.580 ^L^ 0.400 ^L^ 0.560 ^L^ 0.526 ^L^						
**Kinetics**								
**Hip kinetics**			**Knee kinetics**			**Ankle and foot kinetics**		
**FO effect (sub-phase)**	**S**	**ES (I/L)**	**FO effect (sub-phase)**	**S**	**ES (I/L)**	**FO effect (sub-phase)**	**S**	**ES (I/L)**
No information			↑Max external rotation moment ↑External rotation impulse (LP) ↑Internal ABD moment ↑Max internal ABD moment ↑External ADD moment ↑Max external ADD moment ↑External ADD moment integral ↓Max patellofemoral force ↓Patellofemoral force per mile ↓Max patellofemoral stress	62, 62 54 59 78 69 69 68 68 68 68	• ^I^, • ^L^ 0.620 ^L^ 0.091 ^I^ 0.400 ^I^ 0.283 ^I^ 0.289 ^I^ 0.320* ^L^ 0.410* ^L^ 0.370* ^L^ 0.420* ^L^	↓Max internal inversion moment ↓Inversion impulse (LP) ↓Negative work	62, 78, 62, 54 62, 62, 54 78	• ^I^, 0.460 ^I^, • ^L^, 0.670 ^L^ • ^I^, • ^L^, 0.760 ^L^ 0.660 ^I^

Immediate and long-term (≥ 3 weeks) effects of foot orthosis (FO) compared to not wearing FO on running kinematics and kinetics. Only significant results (p < 0.05) are presented. Kinematic and kinetic effects during stance phase are reported, and the subphase-specific occurrence is in parenthesis if available (*i.e*., initial contact (IC), loading phase (LP), and propulsion phase (PP)). For the ankle and foot kinematics, Study number (S) is based on the reference number within the text. Effect sizes (ES) (Cohen's d or partial eta squared *) are reported respective to the articles' order, if available (• if not) for immediate^I^ and/or long-term effect ^L^. Abbreviations: ABD, abduction; ADD, adduction; FF, maximal (max); forefoot; RF, rearfoot; ROM, range of motion; ↑, increased; ↓, decreased

*Knee and tibia.* For the immediate effects on the knee joint, an increased rotational range of motion (ROM) (ES = 0.150 − very small effect) was reported with PFOs [67] and an increased knee adduction (ES = 0.390 − small effect) [79] was observed, regardless of the CFO that the participant used (*i.e*., with a 4° external rearfoot (RF) medial wedge and an intrinsic forefoot (FF) wedge or inverted between 15° and 25°) [79]. After 4 weeks of habituation with the PFO, one study reported a decreased knee flexion at initial contact (ES = 0.510 − medium effect) and maximal flexion (ES = 0.280 − small effect) [69]. For the immediate effect of FOs on tibial rotation (tibia rotation relative to the fixed foot segment), an increased maximal internal rotation (ES = 0.400 − small effect) was observed [79]. This result was not supported by two other studies that assessed an immediate decreased tibial internal rotation ROM (ES = 0.191 − very small effect) during overground running with PFO [70], and decreased tibial internal rotation ROM (ES = 0.414 − small effect) during treadmill running with CFO following 3–4 weeks of habituation [66]. A decreased maximal tibial internal rotation (ES = 0.480 − small effect) was also observed during running, following a 6-week adaptation period with CFOs, regardless of the shoe midsole hardness (*i.e*., soft, medium, hard) [55].

*Ankle and foot* For the ankle and foot kinematics, three studies observed immediate effects of wearing FOs on ankle dorsiflexion: increased dorsiflexion at initial contact (ES = 0.277 − small effect) [72], increased maximal dorsiflexion (ES = 0.187 − very small effect) [72], increased dorsiflexion range of motion (ES = n/a) [71], and increased maximal dorsiflexion velocity during loading phase (ES = 0.267 − small effect) [70]. These studies were conducted at self-selected speed with PFO [70, 71] or CFO [72]. Immediate and long-term effect (*i.e*., 6 weeks [55, 63] for 2 studies and time not mentioned for one study [62] of FOs were highlighted on RF eversion (*e.g*., the angle between tibia and calcaneus): immediate effect: decreased RF eversion at initial contact (ES = 0.352 − small effect [72] and 0.650 − medium effect [67]), decreased RF eversion range of motion (ES = 0.360 − small effect [67] and 1.334 − very large effect [77]), decreased maximal RF eversion (ES = 0.500 -medium effect [72], 0.810 − large effect [67], and n/a [63] for the studies with available ES) [63, 67, 72], increased maximal RF eversion (ES = 1.010 − very large effect [61], 0.642 − medium effect [62]), decreased mean RF eversion velocity (ES = 0.387 − small effect) [72], and maximal RF eversion velocity (ES = 0.276 − small effect [72], n/a [63], 0.710 − medium effect [67], and 2.247 − very large effect [77]); long-term effect: decreased RF eversion at initial contact (ES = 0.315 − small effect) [62], decreased maximal RF eversion (ES = n/a) [63], decreased maximal RF eversion velocity (ES = 0.740 − medium effect [55] and n/a [63]). Only two studies assessed the effect of FOs on calcaneal eversion, which was defined as the angle between the calcaneus and the floor [77] or the eversion angle of the calcaneus relative to the laboratory coordinate system [55]. One study reported immediate effects of FOs during treadmill running at self-selected speed: decreased calcaneal eversion range of motion (ES = 1.213 − very large effect), and maximal calcaneal eversion velocity (ES = 1.638 − very large effect) [77]. The other study reported a decreased maximal calcaneal eversion (ES = 0.590 − medium effect) during overground running with FOs at self-selected speed, after a 6-week habituation period [55]. Finally, only one study reported immediate effects of FOs on forefoot motion [71], which was highlighted by a decreased rotation range of motion (ES = n/m).

*Coordination* Concerning intralimb coupling, two studies assessed effects of FOs during treadmill running at self-selected speed following six weeks [74], or 3–4 weeks [66] of habituation. One study reported a decreased variability for tibial rotation and calcaneal eversion-inversion ratio during loading phase (ES = 0.580 − medium effect), and decreased variability for knee rotation and RF eversion-inversion ratio during loading phase (ES = 0.400 − small effect) and the entire stance phase (ES = 0.560 − medium effect) [74]. The other study observed an increased phase angle ratio between frontal and transverse motion of the leg (*i.e*., tibial abduction–adduction and tibial rotation) (ES = 0.526 − medium effect) [66].

### The Effect of FOs on Running Kinetics

*Hip* One study investigated the hip kinetic impacts of wearing FOs and reported no effect [60].

*Knee and tibia* Concerning the immediate effects of FOs on the knee joint kinetics, an increased maximal external rotation moment (ES = n/a) with CFO [63], an increased internal abduction moment (ES = 0.091 − very small effect) with SFO [60], an increased external adduction moment (ES = 0.289 − small effect) and maximal external adduction moment (ES = 0.283 − small effect) with PFO [70], and an increased maximal internal abduction moment (ES = 0.400 − small effect) with 2 types of CFO [79] were reported during overground running at self-selected speed (from 3.4 ± 10 to 4 0.0 ± 5% m/s) (see Table 4**, **Fig. 3a). The increased maximal external rotation moment was also observed after a 6-week habituation time (ES = n/a) [63] and was supported by another study that reported an increased external rotation impulse during loading phase (ES = 0.620 − medium effect) while running overground with CFO at self-selected speed after 6 weeks of habituation time, regardless of the midsole composition (*i.e*., hard, medium, or soft) [55]. One study explored 4-week habituation effects of PFO on overground running at self-selected speed, and observed an increased external adduction moment integral (*i.e*., using trapezoidal function) (ES = 0.320 − small effect), a decreased maximal patellofemoral force (ES = 0.410 − small effect), a decreased patellofemoral force per mile (ES = 0.370 − small effect), and a decreased maximal patellofemoral stress (ES = 0.420 − small effect) [69].

*Ankle and foot* Concerning immediate effects of FOs on ankle and foot kinetics, a decreased maximal internal inversion moment (ES = n/a) and a decreased inversion impulse during loading phase (ES = n/a) were observed [63]. The decreased maximal internal inversion moment was also reported in another study (ES = 0.460 − small effect) that also observed a decrease in negative work (ES = 0.660 − medium effect) [79]. The decreased maximal internal inversion moment and inversion impulse during loading phase were also observed after 6 weeks of habituation time with CFO in two studies (decreased maximal internal inversion moment: ES = 0.670 − medium effect [55] and n/a [63], decreased inversion impulse during loading phase: ES = 0.760 − medium effect [55] and n/a [63]).

### The Effect of FOs on Running Plantar Pressure and Ground Reaction Force

*Plantar pressure distribution* All four studies that assessed the effects of FOs on plantar pressure distribution evaluated overground running (mean speed range: 2.2–3.8 m/s for the three of four studies that mentioned the running speed) [56, 59, 72, 78] and noted immediate effects (see Table 5**, **Fig. 3a). An increase in the lateral plantar pressure over the entire loading phase was observed in three studies [56, 59, 72]. More specifically, CFOs increased the maximal lateral balance pressure magnitude of the foot (ES = 0.431 − small effect) and heel (ES = 0.431 − small effect) [72], PFOs increased the maximal pressure (ES = 0.990 − large effect) and the absolute impulse (ES = 1.040 − very large effect) under the 5th metatarsal [56], and PFOs with different combinations of medial arch height (*i.e*., 20 and 24 mm) and FF wedges (*i.e*., medial and lateral 2–4 mm) deviated the trajectory of the center of pressure laterally (ES = n/a) [59]. Moreover, one study reported that running with PFOs decreased the absolute impulse of the midfoot over the entire loading phase (ES = 1.000 − large effect), shifted the plantar pressure distribution laterally at forefoot flat (ES = n/a) and heel-off (ES = n/a), and shifted laterally the center of pressure’s trajectory (ES = 1.380 − very large effect) at forefoot flat [56]. Along with the increase in lateral plantar pressure, one study observed a decrease in the medial plantar pressure, as their PFOs with various correcting elements decreased the medial force–time integrals at the heel (ES = n/a) and under the 2nd metatarsal (ES = n/a) over the entire loading phase [59]. Finally, a decrease in lateral displacement of the center of pressure during propulsion phase (ES = 1.000 − large effect) [56] and overall increase in maximal (ES = 0.980 − large effect) and mean (ES = 0.310 − small effect) plantar pressure [78] were reported with PFOs.

**Table 5:** Immediate and long-term effects of wearing a foot orthosis compared to not doing so on pain and symptoms, muscle electromyography, and plantar pressure and force

Pain and symptoms			Pressure and force		
FO effect (scale used)	S	ES ^I/L^	FO effect (sub-phase)	S	ES ^I/L^
↓ Knee pain (KOOS-PF) ↓ Knee pain (VAS) ↓ Knee pain (NS) ↓ Knee injury symptoms (NS) ↓ Patellofemoral pain (VAS) ↓ Heel pain (VAS) ↓ Achilles tendon pain (NS) ↓ Achilles tendon pain (PES) ↓ Ankle pain grade (NSS) ↓ MTSS symptoms (MTSSS) ↓ Subjective pain (PES) ↓ Overall pain (PES)	68 67 62 62 53 77 61 63 75 56 52 49	0.650* ^L^ 0.667 ^I^ 0.920 ^L^ • ^L^ 0.151 ^L^ 0.650^I^ • ^L^ • ^L^ 1.177 ^I^ 0.800 ^L^ 0.640 ^L^ 0.746 ^L^	↓ Max vertical (IC) ↓ Vertical loading rate (IC) ↓ Max loading rate (IC) ↓ Total contact time ↑ Max lateral heel BP magnitude ↑ Max lateral foot BP magnitude Later max lateral heel BP Later max lateral foot BP Later max medial heel BP Later max medial foot BP Later forefoot flat contact ↑ Max plantar pressure ↑ Mean plantar pressure ↑ Max M5 pressure ↑ Absolute M5 impulse ↓ Medial heel force–time integral ↓ M2 force–time integral ↓ Absolute midfoot impulse Lateral pressure distribution shift (forefoot flat) Lateral pressure distribution shift (heel off) Lateral COP shift (forefoot flat) ↑ Lateral COP deviation ↓ Lateral COP displacement (PP)	62, 62, 54 62, 62 54 55 71 71 71 71 71 71 58 77 77 55 55 58 58 55 55 55 55 58 55	• ^I^, • ^L^, 0.620 ^L^ • ^I^, • ^L^ 0.670 ^L^ 0.990 ^I^ 0.431 ^I^ 0.431 ^I^ 0.510 ^I^ 0.603 ^I^ 0.155 ^I^ 0.155 ^I^ • ^I^ 0.980 ^I^ 0.310 ^I^ 0.990 ^I^ 1.040 ^I^ • ^I^ • ^I^ 1.000 ^I^ • ^I^ • ^I^ 1.380 ^I^ • ^I^ 1.000 ^I^
**Muscle EMG**					
**FO effect (sub-phase)**	**S**	**ES **^**I/L**^			
↑ TA amplitude (LP) ↓ BF amplitude (LP) ↑ PL preactivation amplitude (before IC)	74 74 50	2.315 ^L^ 2.362 ^L^ • ^L^			

Immediate and long-term (≥ 3 weeks) effects of a foot orthosis (FO) compared to not wearing FO on general comfort and symptoms, muscle electromyography (EMG), and plantar pressure and force. Only significant results (*p* < 0.05) are presented. GRF and muscle EMG changes during stance phase are reported, and the subphase-specific occurrence is in parenthesis if available (*e.g*., initial contact (IC), loading phase (LP), and propulsion phase (PP)). Study number (S) is based on the reference number within the text. Effect sizes (ES) (Cohen's d or partial eta squared *) are reported respective to the articles order, if available (• if not) for immediate^I^ and/or long-term effect ^L^. Abbreviations: BP, balance pressure; COP, center of pressure; CWQ, Coppa-Wonca questionnaires; KOOS-PF, Knee injury and osteoarthritis outcome score—patellofemoral scale; MTSS, medial tibial stress syndrome; MTSSS, medial tibial stress syndrome score scale; M2, second metatarsal; M5, fifth metatarsal; NSS, numeric scale; PES, pain experience scale; PL, peroneus longus; SF-36, short-form health survey; TA, tibialis anterior; BF, biceps femoris; VAS, visual analog pain score; ↑, increased; ↓, decreased

*Plantar pressure timing* Immediate effects of FOs were reported: CFOs delayed the maximal medial and lateral balance pressure magnitude of the foot (medial ES = 0.155 − very small effect and lateral ES = 0.603 − medium effect) and the heel (medial ES = 0.155 − very small effect and lateral ES = 0.510 − medium effect) [72], PFOs decreased the total contact time (ES = 0.990 − large effect) [56], and PFOs with various correcting elements delayed the forefoot flat contact (ES = n/a) [59] (see Table 5**, **Fig. 3a).

*Ground reaction force* CFOs with a RF medial wedge during overground running in runners with an overuse knee injury induced an immediate decrease in the maximal vertical impact (ES = n/a) and vertical loading rate (ES = n/a) at initial contact [63] (see Table 5**, **Fig. 3a). After a 6-week habituation period, decreases in maximal vertical impact (ES = n/a [63] and 0.620 − medium effect [55]), vertical loading rate (ES = n/a [63] and n/a [55]), and maximal loading rate (ES = 0.670) were observed [55]. Of note, one study observed these long-term effects regardless of the shoe midsole hardness (*i.e*., soft, medium, hard) [55].

### The Effect of FOs on Running Muscle Activity

Two studies noted changes in EMG activity associated with a FO intervention in runners, both reporting long-term effects of CFOs (see Table 5**, **Fig. 3a) [51, 75]. An increase in EMG signal amplitude of the tibialis anterior (ES = 2.315 − very large effect) and a decrease in EMG signal amplitude of the biceps femoris (ES = 2.362 − very large effect) was reported during the loading phase [75]. Also, an increase in peroneus longus preactivation EMG signal amplitude was reported before initial contact [51].

### The Effect of FOs on Pain and Symptoms

Among the 12 studies that investigated the effect on pain and symptoms, 11 studies noted a positive effect of FOs on the pain and symptoms associated with lower-limb running injuries (see Table 5**, **Fig. 3b) [50, 53, 54, 57, 62–64, 68, 69, 76, 78]. SFOs had a positive effect on AKP, as shown by the immediate effect (ES = 0.667 − medium effect) [68] of SFOs with a RF medial wedge on pain decrease. This positive effect was also reported on PFPS after a 6-week habituation time with SFOs, with either a full-length medial or lateral wedge (ES = 0.151 − very small effect) [54]. Concerning PFOs, a long-term (4 weeks) effect on PFPS (ES = 0.650 − medium effect) was noted, as shown by the decreased Knee injury and Osteoarthritis Outcome Score (Patellofemoral scale) [69]. A long-term effect (6 weeks) of wearing CFOs with a RF medial wedge on the reduction of general knee pain (ES = n/a) and injury symptoms (ES = n/a) was also reported [63]. One study reported long-term (6 weeks) (ES = 0.800 − large effect) reduction of MTSS symptoms in runners when PFOs were worn during running in addition to exercise therapy, shockwave, and ice [57]. The only study that examined traumatic running-related injury reported that CFOs had an immediate effect on ankle pain (ES = 1.177 − very large effect) in a population who experienced an inversion sprain 6 weeks prior [76]. Concerning heel injuries, wearing CFOs, either with a medial wedge (ES = n/a) [62] or a FF lateral wedge (ES = n/a) [64], reduced pain in runners with chronic AT after 4 weeks or more of habituation. Concerning foot injury, PFOs with a RF medial wedge had an immediate effect (ES = 0.650 − medium effect) on pain reduction in runners with PF [78]. Lastly, CFOs with a FF lateral wedge reduced pain (ES = 0.640 − medium effect) after 8 weeks of habituation in participants with various running-related overuse injuries (*e.g*., PFPS, MTSS, AT, PF) [53]. These results were supported by another study that noted a reduction in pain after 16 weeks (ES = 0.746 − medium effect) and 52 weeks (ES = 0.718 − medium effect) of wearing CFOs in runners with lower-leg running-related overuse injuries (*e.g*., MTSS, AT, PF) [50].

## Discussion

### Summary

This scoping review aimed to describe the immediate and long-term effects of wearing FOs in a symptomatic population on running biomechanics (kinematics, kinetics, plantar pressure), muscle activity, and pain and symptoms, and to identify the factors influencing these effects. Five main findings warrant highlighting. First, wearing FOs while running is related to an immediate and a long term decrease in pain and symptoms of frequent overuse running injuries, especially knee injuries (PFPS, AKP, MTSS, AT, PF). Second, wearing FOs while running decreases eversion at the foot/ankle complex (*e.g*., decrease RF eversion range of motion, decrease ankle inversion impulse). Third, wearing FOs while running leads to a more lateral plantar pressure at the heel and forefoot. Fourth, wearing FOs may change running motor control strategies, by increasing ankle/foot complex muscle activity amplitude, but not its temporal feature. Fifth, the added features of the FO are the factors that mostly influence the biomechanical effects of FOs.

### Running Injuries

*Knee injuries* The knee represents the primary location for overuse running injuries [9]. Literature not specific to runners has shown that FOs are effective in the management of common knee overuse injuries (*e.g.,* PFPS, AKP) as they outperform a wait-and-see strategy [80] and sham/flat insoles [81], and are as effective as hip exercises [82]. Similarly, our results have shown that FOs, regardless of the FO type, are effective in decreasing the pain and symptoms of overuse running knee injuries (*i.e.,* AKP, PFPS, or general knee injury) [54, 63, 69] and are more effective than sham/flat insoles [68]. However, Bonacci et al. [52] noted that wearing FOs during running was less effective than a gait retraining protocol with minimalist shoes for runners with PFPS. This finding aligns with a recent systematic review and meta-analysis indicating that wearing FOs was less effective than treatments such as physiotherapy and gait retraining in non-runners with PFPS [81]. This suggests that FOs should not be the primary treatment option for runners experiencing PFPS or AKP. Instead, FOs might be more effectively utilized as part of a comprehensive treatment approach, in line with recommendations by an expert consensus on patellofemoral pain [83].

Hip muscle stabilization has been identified as a risk factor for overuse knee injuries in runners [84]. In clinical practice, a longstanding rationale for FOs prescription in cases of PFPS and AKP is the belief that by reducing excessive foot pronation, they could potentially decrease the knee valgus quadriceps (Q) angle, thereby reducing lateral stress on the patellofemoral joint [85–87]. Another possible mechanism proposed by Hertel et al. (2005) suggests that, through the enhanced muscle activity of the vastus medialis and gluteus medius, FOs may reduce the excessive lateral movement of the patella [88]. However, some systematic reviews on the effects of FOs in individuals with PFPS have questioned these mechanisms, reporting limited or no effects of FOs on knee kinematics [85], patellofemoral joint load [89], and thigh muscles activation [85]. Similar findings were reported by other systematic reviews involving uninjured runners [39, 41]; however, these later reviews consistently noted a decrease in frontal plane ankle range of motion [41] and internal inversion moment [39]. The results of this scoping review align with the current body of literature by reporting either no evidence [60–63, 65, 72, 74] or conflicting findings [55, 66, 67, 69, 70, 79] regarding knee kinematics and observing no effects on thigh muscle activation [65]. Also, this scoping review observed a decrease in eversion motion at the ankle (RF eversion) and foot (calcaneal eversion), as well as a decrease in internal ankle inversion moments and impulses [55, 63, 67] in runners with an overuse knee injury. A lateral deviation of the center of pressure during running with FOs in participants with an overuse knee injury was also reported, which was theorised by the authors to reflect the decrease in internal ankle inversion moments through the alteration of the lever arm of the ground reaction forces [59]. In the same vein, the results of this scoping review indicate that wearing FOs during running leads to a greater knee internal abduction [60], and external adduction [69, 70] moments in runners with PFPS, which suggest a reduction of internal load within the knee. Overall, our results suggest that the redistribution of the load within the knee joint structures may explain the therapeutic benefits of FOs in cases of runners with overuse knee injuries such as PFPS and AKP.

*Tibia injuries* There is evidence suggesting that FOs can be effective as part of a multimodal treatment plan for active individuals with MTSS [90, 91] and as a standalone treatment in runners with MTSS according to survey-based retrospective studies [92, 93]. Our results reinforce that FOs can be therapeutically effective in runners with MTSS when used in conjunction with other treatment modalities, especially with the findings of Naderi et al. [57], whose study received a score of 100% in our methodological quality assessment [57] (see Table 5). Indeed, they reported that FOs decrease MTSS symptoms when used as part of a comprehensive treatment plan including exercise, shockwave, and ice therapy [57].

FOs are proposed to benefit runners with MTSS by reducing eversion motion of the foot–ankle complex [94, 95], redistributing plantar pressures (lateral shift) [94], and reducing soleus muscle activation [95], all of which are recognized as mechanisms that may reduce risk factors for MTSS [94, 95]. The findings of this scoping review suggest that wearing FOs in runners with MTSS [56] or various lower-limb injuries [72] induces a lateral shift of the plantar pressures [56, 72] and the trajectory of the center of pressure [72]. It has been unanimously suggested that these adaptations resulted from a decrease in foot eversion, thereby supporting these proposed mechanisms [56, 72]. Considering that 72% of participants included in one of these studies had pronated feet [56], it could be suggested that targeting a lateral shift in plantar pressures with FOs in runners with pronated feet suffering from MTSS might reduce the pain and symptoms associated with their condition.

*Foot and heel injuries* The only study specific to a population with PF that was included in this scoping review observed that FOs immediately decreased pain during running and were as effective as anti-pronation tape [78]. These results are in line with systematic reviews with meta-analyses, not specific to runners, reporting that FOs had low to moderate effect in managing the pain and symptoms associated with PF [15, 96], and were as effective as other conservative treatments [97]. Nonetheless, the results of this scoping review highlight the absence of longitudinal studies (n = 0) to recommend FOs in the management of PF in runners. Only one study focused on AT and reported that wearing FOs during running was as effective as a standard physiotherapy intervention (including an eccentric exercise program), and better than no treatment [64]. However, this study lacked essential measures to mitigate bias, notably the absence of participant blinding and failure to assess intervention compliance [64]. Therefore, the results of this scoping review only add to the limited evidence concerning the effectiveness of wearing FOs as a standalone treatment for reducing AT [98, 99] and PF [15, 96] pain and symptoms in the general population, and further research is needed to compare their effectiveness to other treatments in a population of symptomatic runners.

In the current literature, the hypothesis put forth for using FOs in the management of foot and heel injuries such as AT and PF is that they may reduce RF and calcaneal eversion in a population with excessive foot pronation which should decrease the bending stress within the Achilles tendon [99] and decrease the strain on the plantar fascia [100]. Indeed, when wearing FOs, a reduction in maximal calcaneal and RF eversion magnitude or velocity has been commonly reported by the included studies regardless of the injury [55, 63, 67, 72, 77], suggesting a reduction of RF and calcaneal eversion with FOs. However, only the two studies that specifically focused on runners with AT injury observed an increase in maximal RF eversion angle and range of motion, which is more representative of a different motor pattern and soft tissue loading than a decrease in eversion [61, 62]. It is noteworthy that all participants were the same in both of these studies, which were conducted by the same first author. Another hypothesis put forth for using FOs in the management of AT is that they may normalize the neuromotor activity of the triceps surae, which could impose a more homogeneous stress on the Achilles tendon [101]. However, Wyndow et al. (2013) observed no significant difference in relative offset timing between the soleus and the gastrocnemius lateralis when running with and without FOs in runners with AT injury [58], suggesting that the stress imposed on the Achilles tendon and the temporal dynamics of calf muscles may remain similar. Furthermore, the study included in this scoping review that focused on runners with PF showed that wearing FOs induced no significant change in medio-lateral pressures, suggesting that the foot motion and stress on the plantar fascia may be similar [78]. However, they acknowledged that most of their participants had a neutral foot alignment (82%), which might have constrained the observed effects of FOs. Together, these findings highlight the need for further research to clarify the underlying mechanisms driving the clinical effectiveness of wearing FOs in runners suffering from foot or heel injuries such as AT and PF.

### Clinical Implications and Recommendations for Future Research

This scoping review aimed to identify factors that may influence the effects of FOs to assist clinicians in their decision-making process and guide future research. Based on our review, researchers and clinicians may need to consider factors such as the FO type and its added features, the foot posture, and the adaptation period. The key factors identified are discussed in the following sections.

*FO type and added features* The added features refer to components such as wedges and arch supports added to the FO. Only one study directly compared two different types of FOs such as SFO with added features and CFO, but found no significant difference in the excessive foot eversion of runners when wearing a SFO with an arch support and a 5° RF wedge, and a CFO [77]. Nevertheless, insufficient details were provided regarding their CFO prescription and its added features, which does not enable determination of whether CFOs should be preferred to SFOs with added features. Since CFOs might be more cost-effective [41], future research should consider comparing SFOs and PFOs to CFOs for symptomatic runners. Moreover, 7 out of the 16 included studies that assessed the effects of CFOs were rated as of low methodological quality. The assessment of the effects of the FO added features was also limited by lack of information given by the authors on the FO components. However, it is notable that all studies reporting a reduced RF and calcaneal eversion motion [55, 63, 67, 72, 77], or internal ankle inversion moment or impulse [55, 63, 79], were conducted with FOs featuring a RF medial wedge. Therefore, the results indicate that a RF medial wedge should be used if the aim of FOs is to decrease RF and/or calcaneal eversion motion and moments in injured runners. This recommendation aligns with Moisan et al. [27], who suggested that FOs should be designed with stronger pronation-reducing elements when used in high-impact tasks or activities such as running. Indeed, in an 8-week intervention study, wearing FOs designed with features aiming at reducing foot pronation was related to sensorimotor adaptations such as an increased peroneus longus preactivation amplitude, which increase ankle stability at initial contact [51].

To facilitate comparisons between studies, FO type and added features should be described in a standardized manner, following the proposed criteria: the type of FO based on a common classification (*e.g*., SFO, PFO, CFO), the full-description of the added features with details (*e.g*., 20-mm medial arch support, 4° RF extrinsic medial wedge), the criteria justifying the use of added features when they are not uniformly applied for all participants, the customization method (*e.g*., weight bearing heat molding, non-weight bearing neutral cast), the length of the FO (*e.g*., full length, 3/4 length), the heel cup depth, and the material and rigidity of each element of the FO (*e.g*., 3-mm shell of polypropylene shore A50 with a 3-mm neoprene cover shore A20, and extrinsic RF medial wedge of ethylene–vinyl acetate shore A65). Finally, authors should use sham/flat insoles as a controlled and blinded condition for participants, which is crucial for generating high-quality evidence by minimizing the placebo effect [102]. For instance, 26 of the 30 included studies did not blind the participants to the intervention, which highlights the lack of blinding in the current literature.

*Foot posture* The results of this scoping review suggest that runners suffering from a knee overuse injury with pronated feet [68] and neutral feet [69] may experience knee pain and symptom relief when wearing FOs during running. Similarly, Matthews et al. (2020) observed no association between midfoot width mobility and treatment outcome when evaluating FOs as a treatment for PFPS in a non-runner specific population [82]. Thus, foot posture appears to not influence the effect of FO treatment outcomes in a population with knee overuse injuries, which suggests that other quantitative biomechanical measurements may play a more significant role in determining the efficacy of FOs in such cases. Thus, the foot morphology (*e.g*., supination resistance, navicular drop) and additional dynamic quantitative biomechanical measurements (*e.g*., plantar pressure) of the participants should be reported in future studies to extend conclusions to other injuries and help clinicians to decide which patients might benefit from FOs while running.

*Adaptation period* The findings of this scoping review suggest that pain and symptoms relief are likely when wearing FOs during running, either with (long-term effect) [53, 54, 57, 62–64, 69] or without (immediate effect) [68, 76, 78] an adaptation period. The FOs' effects were also observed on kinematic and kinetic pronation reduction immediately after orthotic dispense [63, 67, 72, 77, 79], and after an adaptation period [55, 62, 63]. The two included studies that specifically compared the immediate and long-term effects of wearing FOs on the biomechanical parameters during running noted no significant differences induced by an adaptation period [51, 63]. Once more, these findings suggest that FOs have the potential to immediately redistribute loads to other structures during running, an effect that seems to also endure in the long term. These results also imply that the biomechanical effects of FOs can be studied immediately after orthotic dispense.

### Limitations

Limitations of this scoping review need to be recognized. First, the literature search was restricted to publications in English and French to ensure a comprehensive understanding of the content and precise extraction of relevant information. Second, the included studies were published between 1992 and 2023. Over this period, running shoes have undergone technological advancements, incorporating functional elements aimed at offering stability and/or enhancing performance. Indeed, these features may influence the effects of FOs reported by the 16/30 studies that were conducted with the participants using their preferred running shoes [50, 52, 56, 57, 61, 62, 64–66, 68–70, 76–78]. However, not controlling the shoes worn enhances the ecological validity of the assessment, by reflecting the real-world scenarios where individuals typically use their preferred shoes for running.

## Conclusion

This scoping review provides crucial recommendations for future research on FOs in injured runners. Specifically, it emphasizes the need for standardized methods in describing FOs, encompassing the type, detailed added features, customization techniques, length, heel cup depth, and material properties of each component. Additionally, it underscores the importance of considering participant characteristics, such as foot morphology, and advocates for high-quality study designs, including the use of sham/flat insoles for control and blinding purposes. Furthermore, to advance current knowledge, comparison between different types of FOs (*e.g*., comparing SFO and PFO to CFO) is encouraged. For clinical practice, this scoping review provides valuable insights to guide the prescription and design of FOs. Overuse running injuries are related to an imbalance between the repetitive load applied to a structure and its adaptive capacity. This scoping review indicates that FOs can redistribute loads onto other structures (*e.g.,* by including a medial wedge to reduce rearfoot and/or calcaneal motion and moments), thus leading to an immediate reduction in pain and potentially treating the injury. However, injuries have multifactorial and complex causes (both intrinsic and extrinsic) and cannot therefore be entirely attributed to biomechanical risk factors of movement. Thus, integrating FOs into a comprehensive treatment plan is suggested to yield better results compared to standalone first-line treatments. Nonetheless, further research is needed to explore the optimal integration of FOs into injury-specific treatment plans.

## Supplementary Information

Additional file 1.

## Data Availability

Data can be provided on reasonable request. Supplemental material associated with this article can be found in the online version.

## Abbreviations

AKP: Anterior knee pain
AS: Ankle sprain
AT: Achilles tendinopathy
CFO: Customized foot orthosis
EMG: Electromyography
ES: Effect size
FF: Forefoot
FOs: Foot orthoses
ITS: Iliotibial band syndrome
MTSS: Medial tibial stress syndrome
PF: Plantar fasciitis
PFO: Prefabricated foot orthosis
PFPS: Patellofemoral pain syndrome
RF: Rearfoot
ROM: Range of motion
SD: Standard deviation
SFO: Simple foot orthosis
