# Supplementary material for: Can Foot Orthoses Benefit Symptomatic Runners? Mechanistic and Clinical Insights Through a Scoping Review
Source: Sports Med Open. 2024 Oct 4;10:108. doi: 10.1186/s40798-024-00774-w (PMC11452579; doi:10.1186/s40798-024-00774-w)
Supplement: Supplementary file 1 — Additional file 1. [file 40798_2024_774_MOESM1_ESM.docx]

**Supplementary materials**

**Supplementary material 1.** PICO question and details

| **Population (P)** | **Intervention (I)** | **Comparison (C)** | **Outcome (O)** |
| --- | --- | --- | --- |
| Symptomatic and/or injured adult runners | Use of foot orthoses (FOs) during running | If available. No foot orthoses or alternative interventions excluding simultaneous interventions (e.g., FOs and physiotherapy) or different footwear types (e.g., military boots) | Immediate or long-term effects on running kinematics, kinetics, EMG, plantar pressure, force, pain, comfort, and injury symptoms |

Question

In symptomatic runners, how does the use of foot orthoses during running, compared to no foot orthoses or alternative treatments (excluding simultaneous interventions), affect immediate or long-term outcomes in running kinematics, kinetics, EMG, plantar pressure, force, pain, comfort, and injury symptoms?


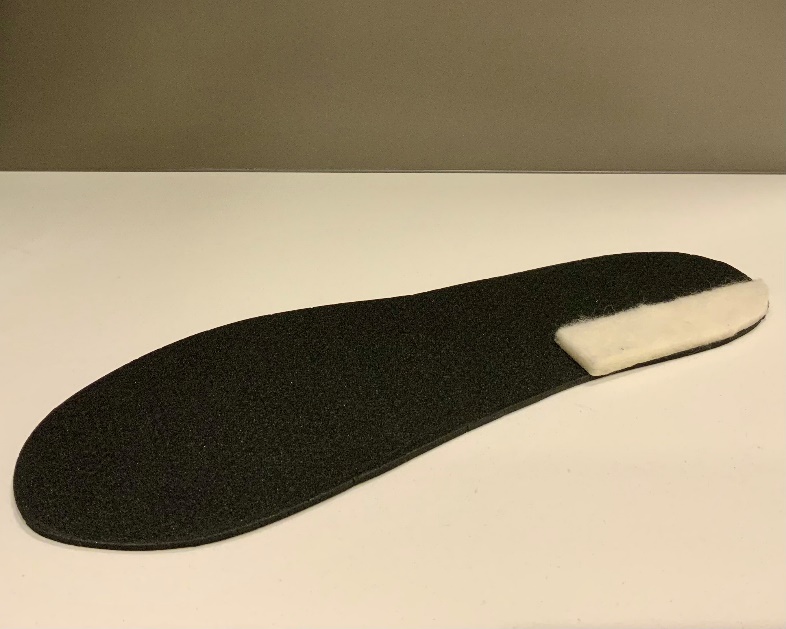

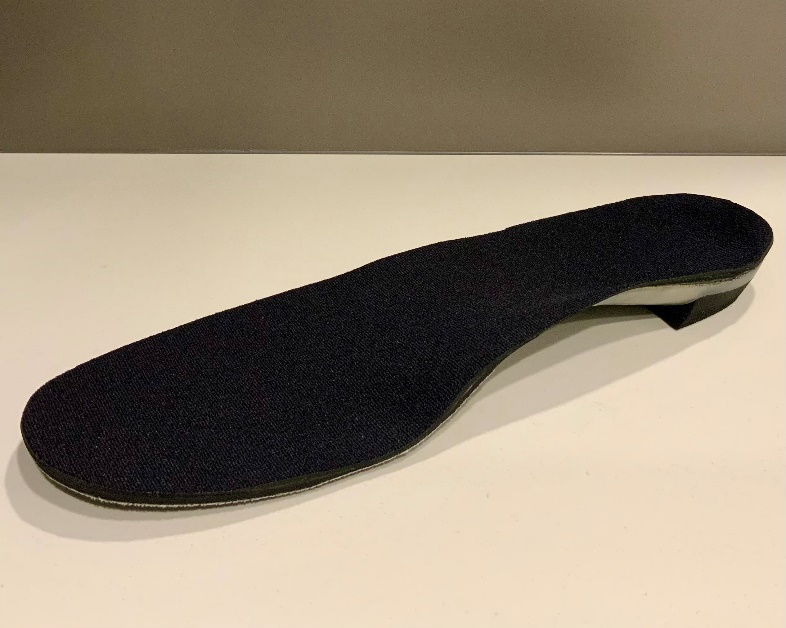

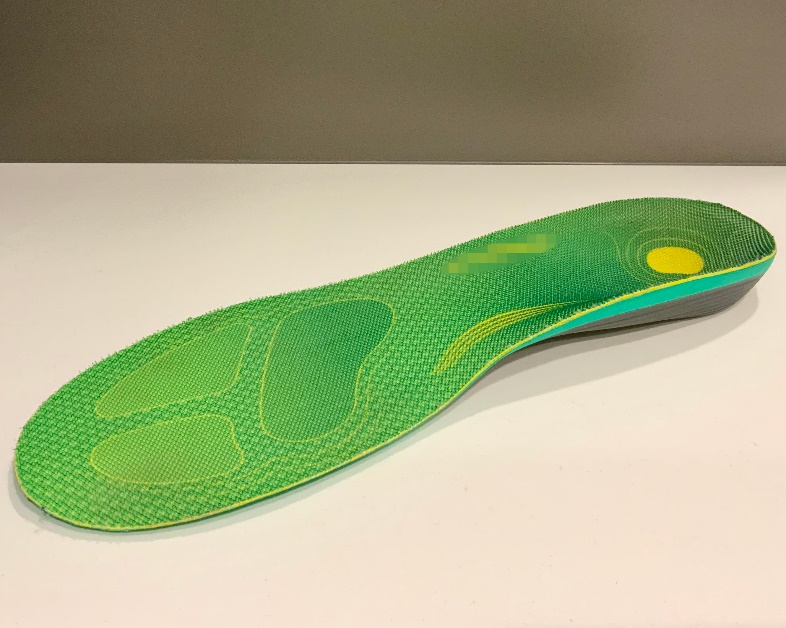


**A**

**B**

**C**

**Supplementary material 2.** Example of a common simple foot orthosis (A), prefabricated foot orthosis (B), and custom foot orthosis (C).

**Supplementary material 3.** Detailed description of the foot orthosis evaluated by the included studies

| **Study** | **Assessed conditions** FO type worn, added features & specifications | **FO customization method** | **FO specification** specification (material, hardness) |
| --- | --- | --- | --- |
| Andreasen et al. (2013) [50] | SG1: Personal footwear SG2: CFO, n/a | SG2: moulded and wedged individually | SG2: FO general (EVA, A35) |
| Baur et al. (2011) [51] | SG1: Neutral footwear SG2: CFO, 3-mm FF lateral wedge and 25-mm arch support | SG2: Dynamic barefoot plantar pressure distribution | SG2: full-length FO with bowl-shaped heel (EVA, semi-rigid) |
| Boldt et al. (2013) [60] | SG: 1- Study footwear 2- SFO, 6° full-length medial wedge | None | 2- extrinsic wedge (EVA, A65), standardized shoe sock liner (n/m, n/m) |
| Bonacci et al. (2018) [52] | SG:  1- Personal footwear 2- PFO, 6° medial wedge | 2- Optimal comfort: Heat molding, wedge, and/or heel raise | 2- 3/4 or full-length FO (EVA, A52-A75) |
| Dixon & McNally (2008) [72] | SG: 1- Study footwear 2- CFO, n/a | 2- Low, normal, or high arch based on dynamic arch index and medial wedge based on individual need | 2- full-length FO with a base layer (EVA, A60) and a top layer (EVA, A40) |
| Donoghue et al. (2008) [61] | SG:  1- Personal footwear 2- CFO, medial wedge < 10° | n/m | 2- FO general (EVA, n/m) |
| Donoghue et al. (2008) [62] | SG:  1- Personal footwear 2- CFO, medial wedge < 10° | 2- NWB neutral cast | 2- FO general (EVA, n/m) |
| Ferber et al. (2005) [73] | SG:  1- Study footwear 2- CFO, 4° RF intrinsic medial wedge 3- CFO, inverted | 2- NWB neutral cast 3- NWB neutral cast and inverted depending on foot morphology and symptoms | 2- shell with intrinsic FF wedge (graphite, n/m) 3- 15° or 25° inverted shell (graphite, n/m) |
| Hirschmüller et al. (2011) [53] | SG1: Neutral footwear  SG2: CFO, FF lateral wedge | SG2: Dynamic barefoot plantar pressure distribution | SG2: full-length FO with bowl-shaped heel (EVA, semi-rigid) |
| Lewinson et al. (2015) [54] | SG1: SFO, 6-mm full-length medial wedge  SG2: SFO, 3-mm full-length lateral wedge  (Study footwear) | SG1 and SG2: Foot size | SG1 and SG2: full-length insole (3-mm EVA, A60) and extrinsic wedge (EVA, A60) |
| MacLean et al. (2008) [63] | SG:  1- Study footwear 2- CFO, 5° RF intrinsic medial wedge | 2- NWB neutral cast and body weight | 2- shell with 18-mm deep heel cup (3-mm copolymer or polypropylene, semi-rigid), extrinsic RF stabilizer (EVA, n/m), full-length cover (EVA, n/m) |
| MacLean et al. (2009) [55] | SG: With or without: CFO, 5° RF intrinsic medial wedge 1- Soft shoe midsole 2- Medium shoe midsole 3- Hard shoe midsole | SG: NWB neutral cast and body weight | CFO- shell with 18-mm deep heel cup (3-mm copolymer or polypropylene, semi-rigid), extrinsic RF stabilizer (EVA, n/m), full-length cover (EVA, n/m) 1- shoe midsole (n/m, C40) 2- shoe midsole (n/m, C55) 3- shoe midsole (n/m, C70) |
| MacLean et al. (2010) [74] | SG: 1- Study footwear 2- CFO, 5° RF intrinsic medial wedge | 2- NWB neutral cast and body weight | 2- shell with 18-mm deep heel cup (3-mm copolymer or polypropylene, semi-rigid), extrinsic RF stabilizer (EVA, n/m), full-length cover (EVA, n/m) |
| Mayer et al. (2007) [64] | SG1: Personal footwear SG2: CFO, FF lateral wedge, arch support | SG2: Dynamic barefoot plantar pressure distribution | SG2: full-length FO with bowl-shaped heel (EVA, semi-rigid) |
| Mills et al. (2012) [65] | SG1 and SG2:  1- PFO, n/m 2- PFO, n/m 3- PFO, n/m 4- sham/flat insole  (Personal footwear) | None | SG1 and SG2:  1- full-length FO (EVA, A75) 2- full-length FO (EVA, A60) 3- full-length FO (EVA, A52) 4- 3-mm full length insole (EVA, A52) |
| Naderi et al. (2019) [56] | SG: 1- Personal footwear 2- PFO, 25-mm peak-height arch support | None | 2- full-length FO with 15-mm deep heel cup (4-mm polypropylene, A50) |
| Naderi et al. (2022) [57] | SG1: sham/flat insole SG2: PFO, 25-mm peak-height arch support  (Personal footwear) | None | SG1: 3/4 insole (4-mm polypropylene, A50) SG2: 3/4 FO with 15-mm deep heel cup (4-mm polypropylene, A50) |
| Nawoczenski et al. (1995) [66] | SG: 1- Personal footwear 2- CFO, n/a | 2- NWB neutral cast and wedged individually | 2- shell (3-mm polypropylene, semi-rigid), cover (3-mm neoprene, A20) |
| Nawoczenski & Ludewig (1999) [75] | SG: 1- Study footwear 2- CFO, n/a | 2- NWB neutral cast and wedged individually | 2- shell with intrinsic FF wedge (3-mm polypropylene, semi-rigid), extrinsic RF wedge (n/m, n/m), cover (3-mm neoprene, n/m) |
| Orteza et al. (1992) [76] | SG: 1- Personal footwear 2- CFO, n/a 3- sham/flat insole | 2- Molded to the neutral subtalar joint position and wedged individually | 2- shell with intrinsic RF wedge (3-mm Aquaplast, semi-rigid), extrinsic FF wedge (n/m, n/m), full-length cover (3-mm Plastazote, n/m) 3- full-length insole (3-mm Plastazote, n/m) |
| Rodrigues et al. (2013) [67] | SG: 1- Study footwear 2- SFO, 4° RF and FF medial wedges | None | 2- extrinsic wedges (rubber, n/m), New Balance insoles (n/m, n/m) |
| Shih et al. (2011) [68] | SG1: sham/flat insole SG2: SFO, 5° RF medial wedge  (Personal footwear) | SG1 and SG2: Foot size | SG1: full-length insole (2-mm Poron, n/m) SG2: full-length insole (2-mm Poron, n/m) and extrinsic wedge (EVA, n/m) |
| Sinclair et al. (2018) [69] | SG:  1- Personal footwear  2- PFO, n/a | SG: Heat molding to the longitudinal arch profile | SG: full-length FO (EVA, A30) |
| Sinclair & Butters (2021) [70] | SG: 1- Personal footwear 2- PFO n/a | 2- Heat molding to the longitudinal arch profile | 2- full-length FO 6-mm deep heel cup (EVA, A30) |
| Stell & Buckley (1998) [77] | SG: 1- Personal footwear 2- CFO, n/a 3- SFO, 5° RF wedge and arch support | 2- NWB neutral cast 3- Arch filled according to profile | 2- shell with intrinsic RF and FF wedges (EVA, high density) 3- full-length insole (1.5-mm EVA, n/m), extrinsic RF wedge (EVA, n/m) covered (1.5-mm Poron, n/m), filled arch support (Plastazote, n/m) |
| Van Lunen et al. (2011) [78] | SG: 1- Personal footwear 2- PFO, 6° RF medial wedge | None | 2- 1/2 length FO with a 15-mm heel cup (n/m, n/m) |
| Williams III et al. (2003) [79] | SG:  1- Study footwear 2- CFO, 4° RF medial wedge 3- CFO, inverted | 2 and 3- impression n/m 2- wedged in FF individually 3- inversion according to calcaneal stance position | 2- shell with intrinsic FF wedge (graphite, n/m), extrinsic RF wedge (n/m, n/m) 3- 15° or 25° inverted shell (graphite, n/m) |
| Wyndow et al. (2013) [58] | SG: 1- Study footwear 2- PFO, n/a | None | 2- shell (polypropylene, semi-rigid), full-length cover (3-mm sponge, n/m) |
| Zhang et al. (2022) [59] | SG:  1- Study footwear 2- PFO, 4-mm medial FF wedge, 20-mm arch support 3- PFO, 2-mm medial FF wedge, 20-mm arch support 4- PFO, 20-mm arch support 5- PFO, 2-mm lateral FF wedge, 20-mm arch support 6- PFO, 4-mm lateral FF wedge, 20-mm arch support 7- PFO, 4-mm medial FF wedge, 24-mm arch support 8- PFO, 2-mm medial FF wedge, 24-mm arch support 9- PFO, 24-mm arch support 10- PFO, 2-mm lateral FF wedge, 24-mm arch support 11- PFO, 4-mm lateral FF wedge, 24-mm arch support | None | 2 to 11- 3/4 length shell with varying extrinsic FF wedges and arch support (4-mm 3D printed polyurethane, n/m), full-length cover (thin fabric, n/m) |
| Zhang & Vanwanseele (2023) [71] | SG:  1- Study footwear 2- PFO, 4-mm medial FF wedge, 20-mm arch support 3- PFO, 2-mm medial FF wedge, 20-mm arch support 4- PFO, 20-mm arch support 5- PFO, 2-mm lateral FF wedge, 20-mm arch support 6- PFO, 4-mm lateral FF wedge, 20-mm arch support 7- PFO, 4-mm medial FF wedge, 24-mm arch support 8- PFO, 2-mm medial FF wedge, 24-mm arch support 9- PFO, 24-mm arch support 10- PFO, 2-mm lateral FF wedge, 24-mm arch support 11- PFO, 4-mm lateral FF wedge, 24-mm arch support | None | 2 to 11- 3/4 length shell with varying extrinsic FF wedges and arch support (4-mm 3D printed polyurethane, n/m), full-length cover (thin fabric, n/m) |

Abbreviations: CFO, custom foot orthosis; EVA, ethylene vinyl acetate; FF, forefoot; FO, foot orthosis; n/a, not applicable; n/m, not mentioned; NWB, non-weight bearing; PFO, prefabricated foot orthosis; RF, rearfoot; SG, symptomatic group; SFO, simple foot orthosis.
